# Supplementary material for: Compact IF2 allows initiator tRNA accommodation into the P site and gates the ribosome to elongation
Source: Nat Commun. 2022 Jun 13;13:3388. doi: 10.1038/s41467-022-31129-2 (PMC9192638; doi:10.1038/s41467-022-31129-2)
Supplement: Supplementary file 1 — Supplementary Information [file 41467_2022_31129_MOESM1_ESM.pdf]

Supplementary Information for

Compact IF2 allows initiator tRNA accommodation into the P site and gates the ribosome to elongation

Ritwika S. Basu[1], Michael B. Sherman[2,3] & Matthieu G. Gagnon[1,2,3,4,*]

[1] Department of Microbiology and Immunology, University of Texas Medical Branch, Galveston, Texas 77555, USA.

[2] Department of Biochemistry and Molecular Biology, University of Texas Medical Branch, Galveston, Texas 77555, USA.

[3] Sealy Center for Structural Biology and Molecular Biophysics, University of Texas Medical Branch, Galveston, Texas 77555, USA.

[4] Institute for Human Infections and Immunity, University of Texas Medical Branch, Galveston, Texas 77555, USA.

*email: magagnon@utmb.edu

Supplementary Discussion

Global 3D variability analysis

To gain further insights into conformational coupling that might exist between IF2, the ribosome and initiator tRNA in this cryo-EM dataset, we performed global 3D variability analysis using non-rotated and rotated ribosome particles (class averages I and II). Sorting of the particles along the 30S subunit rotation trajectory suggests that the conformational landscape of the 70S-IC spans from the state in which the 30S subunit is rotated with IF2-GDPCP in the extended conformation and interacting with initiator tRNA bound in the p/PI state, to the non-rotated state containing IF2-GDP in the compact conformation and fMet-$tRNA_i^{fMet}$ fully accommodated into the P site (Supplementary Fig. 15). This analysis corroborates our findings and suggests that the large domain movements in IF2 are coupled with the degree of 30S subunit rotation and the state of initiator tRNA accommodation into the P site. Similarly, the extra density attributable to the N2 sub-domain of IF2 on the rotated ribosome disappears as the 30S subunit rotates back upon IF2 transitioning to the compact conformation (Supplementary Movie 2). These observations illustrate how the last step of translation initiation is regulated by concerted structural rearrangements in the ribosome, initiator tRNA, and IF2.

Supplementary Table 1. Cryo-EM data collection, refinement and validation statistics

	Structure I-A (70S) (EMDB-26628)	Focused I-A (IF2-GDP) (EMDB-26629) (PDB 7UNQ)	Composite I-A (70S-IC) (EMDB-26630) (PDB 7UNR)
**Data collection and processing**			
Magnification	105,000x	105,000x	105,000x
Voltage (kV)	300	300	300
Electron exposure ($e^{-}/Å^2$)	31	31	31
Defocus range (µm)	–0.2 to –2.1	–0.2 to –2.1	–0.2 to –2.1
Detector	K3 (Gatan)	K3 (Gatan)	K3 (Gatan)
Pixel size (Å)	0.85 (super res. 0.425)	0.85 (super res. 0.425)	0.85 (super res. 0.425)
Symmetry imposed	C1	C1	C1
Initial particle images (no.)	417,757	417,757	417,757
Final particle images (no.)	21,480	21,480	21,480
Map resolution (Å)	2.9	3.4	2.9
FSC threshold	0.143	0.143	0.143
**Refinement**			
Initial model used (PDB code)		3JCJ	3JCJ, 6SPG
Model resolution (Å)		3.5	3.0
FSC threshold		0.5	0.5
Map sharpening *B* factor ($Å^2$)	–21	–77	-
Model composition			
Chains		1	57
Non-hydrogen atoms		3,528	149,329
Protein residues		486	6,501
RNA residues		1	4,605
Ligands: Mg^{2+}/Zn^{2+}		-	630/2
Waters		-	200
B factors ($Å^2$)			
Protein residues		64	49
RNA residues		46	48
Ions		-	28
Waters		-	25
CC_{mask}		0.82	0.85
R.m.s. deviations			
Bond lengths (Å)		0.002	0.004
Bond angles (°)		0.579	0.600
Validation			
MolProbity score		2.1	1.8
Clashscore		7.4	6.5
Rotamer outliers (%)		0	0
Cβ outliers (%)		0	0
Ramachandran plot			
Favored (%)		90.0	92.4
Allowed (%)		9.2	7.2
Disallowed (%)		0.8	0.4

Supplementary Table 1. Continued

	Structure I-B (70S) (EMDB-26631)	Focused I-B (IF2-GDP) (EMDB-26632) (PDB 7UNT)	Composite I-B (70S-IC) (EMDB-26633) (PDB 7UNU)
**Data collection and processing**			
Magnification	105,000x	105,000x	105,000x
Voltage (kV)	300	300	300
Electron exposure (e^{-}/Å^2)	31	31	31
Defocus range (µm)	–0.2 to –2.1	–0.2 to –2.1	–0.2 to –2.1
Detector	K3 (Gatan)	K3 (Gatan)	K3 (Gatan)
Pixel size (Å)	0.85 (super res. 0.425)	0.85 (super res. 0.425)	0.85 (super res. 0.425)
Symmetry imposed	C1	C1	C1
Initial particle images (no.)	417,757	417,757	417,757
Final particle images (no.)	24,614	24,614	24,614
Map resolution (Å)	2.9	3.6	2.9
FSC threshold	0.143	0.143	0.143
**Refinement**			
Initial model used (PDB code)		3JCJ	3JCJ, 6SPG
Model resolution (Å)		3.7	3.0
FSC threshold		0.5	0.5
Map sharpening *B* factor (Å^2)	–23	–91	-
Model composition			
Chains		1	57
Non-hydrogen atoms		3,270	149,261
Protein residues		472	6,487
RNA residues		1	4,605
Ligands: Mg^{2+}/Zn^{2+}		-	607/2
Waters		-	201
B factors (Å^2)			
Protein residues		41	46
RNA residues		34	44
Ions		-	23
Waters		-	22
CC_{mask}		0.75	0.89
R.m.s. deviations			
Bond lengths (Å)		0.002	0.006
Bond angles (°)		0.559	0.667
Validation			
MolProbity score		1.9	1.8
Clashscore		5.9	6.0
Rotamer outliers (%)		0	0
Cβ outliers (%)		0	0
Ramachandran plot			
Favored (%)		90.5	91.6
Allowed (%)		9.1	8.0
Disallowed (%)		0.4	0.4

Supplementary Table 1. Continued

	Structure II-A (70S-IC) (EMDB-26634) (PDB 7UNV)	Structure II-B (70S-IC) (EMDB-26635) (PDB 7UNW)	Focused N2 sub-domain (EMDB-26553) (PDB 7UIU)
**Data collection and processing**			
Magnification	105,000x	105,000x	105,000x
Voltage (kV)	300	300	300
Electron exposure ($e^{–}$/Å^2)	31	31	31
Defocus range (µm)	–0.2 to –2.1	–0.2 to –2.1	–0.2 to –2.1
Detector	K3 (Gatan)	K3 (Gatan)	K3 (Gatan)
Pixel size (Å)	0.85 (super res. 0.425)	0.85 (super res. 0.425)	0.85 (super res. 0.425)
Symmetry imposed	C1	C1	C1
Initial particle images (no.)	417,757	417,757	417,757
Final particle images (no.)	73,337	98,715	123,146
Map resolution (Å)	2.7	2.6	2.8
FSC threshold	0.143	0.143	0.143
**Refinement**			
Initial model used (PDB code)	3JCJ, 6SPG	3JCJ, 6SPG	-
Model resolution (Å)	2.8	2.8	3.3
FSC threshold	0.5	0.5	0.5
Map sharpening *B* factor (Å^2)	–28	–30	–68
Model composition			
Chains	57	57	1
Non-hydrogen atoms	149,601	149,947	607
Protein residues	6,533	6,533	85
RNA residues	4,604	4,604	-
Ligands: Mg^{2+}/Zn^{2+}	595/2	796/2	-
Waters	295	352	-
B factors (Å^2)			
Protein residues	50	49	62
RNA residues	52	52	-
Ions	32	32	-
Waters	25	26	-
CC_{mask}	0.86	0.86	0.84
R.m.s. deviations			
Bond lengths (Å)	0.003	0.005	0.004
Bond angles (°)	0.587	0.642	0.563
Validation			
MolProbity score	1.8	1.8	1.5
Clashscore	6.8	6.3	3.4
Rotamer outliers (%)	0	0	0
Cβ outliers (%)	0	0	0
Ramachandran plot			
Favored (%)	92.9	92.8	95.2
Allowed (%)	6.7	6.9	4.8
Disallowed (%)	0.4	0.3	0

Supplementary Table 2. Methylated nucleotides modeled in the 16S and 23S rRNAs of the *P. aeruginosa* 70S initiation complex

		rRNA modification	*E. coli* K12 methyltransferase	*P. aeruginosa* PAO1 methyltransferase	% identity	Density for modification	Reference
***E. coli* K12**	***P. aeruginosa* PAO1**						
**16S rRNA**							
G527	G521	m^7G	RsmG	PA5564	53	yes	[1]
G966	G960	m^2G	RsmD	PA0370	50	yes	[2]
C967	C961	m^5C	RsmB	PA0017	51	yes	[3,4]
G1207	G1201	m^2G	RsmC	PA4627	41	yes	[5]
C1402	C1396	m^4Cm [a]	RsmH<br>RsmI	PA4420<br>PA4422	56<br>61	yes	[6-8]
C1407	C1401	m^5C	RsmF	-	-	no	[8,9]
U1498	U1492	m^3U	RsmE	PA0419	46	yes	[8,10]
G1516	G1510	m^2G	RsmJ	PA3680	48	yes	[8,11]
A1518	A1512	$m^6{}_2A$	RsmA	PA0592	54	yes	[8,12,13]
A1519	A1513	$m^6{}_2A$	RsmA	PA0592	54	yes	[8,12,13]
**23S rRNA**							
G745	G735	m^1G	RlmA	PA1191	42	yes	[14]
U747	U737	m^5U	RlmC	-	-	no	[15]
A1618	A1608	m^6A	RlmF	PA3840	56	yes	[16]
G1835	G1822	m^2G	RlmG	PA4617	45	yes	[17]
ψ1915	ψ1902	$m^3\psi$	RlmH	PA4004	56	yes (modelled as m^3U)	[8,18,19]
U1939	U1926	m^5U	RlmD	PA0933	39	yes	[8,15,20]
C1962	C1949	m^5C	RlmI	PA0354	35	yes	[8,21]
A2030	A2017	m^6A	RlmJ	PA5019	42	yes	[22]
G2069	G2056	m^7G	RlmKL	PA3048	47	yes	[23]
G2251	G2238	Gm [a]	RlmB	PA4936	60	yes	[24]
G2445	G2432	m^2G	RlmKL	PA3048	47	yes	[23]
C2498	C2485	Cm [a]	RlmM	PA1563	50	yes	[25]
A2503	A2490	m^2A	RlmN	PA3806	61	yes	[26]
U2552	U2539	Um [a]	RlmE	PA4752	59	yes	[27,28]

[a] Ribose methylated at the 2'OH position

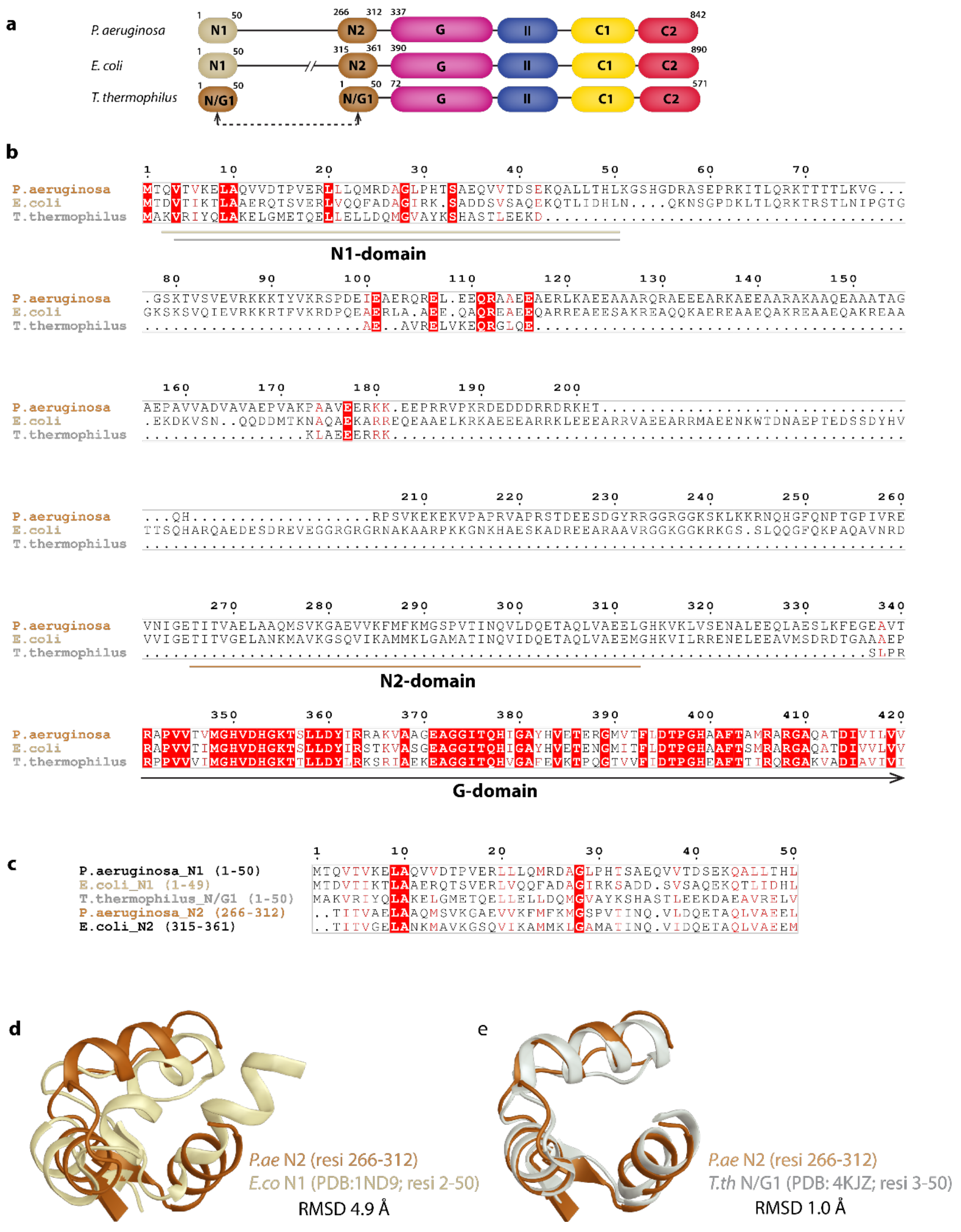

Supplementary Fig. 1. Sequence and structure alignments of the N-terminal domain of IF2. **a** Schematic domain arrangement in IF2. Each domain is colored as in Figure 1. Domain N/G1 in *T. thermophilus* IF2 is aligned with the corresponding sequence repeats within the N-domain in *E. coli* and *P. aeruginosa* IF2. For clarity, we named the region of the N-domain distal from the G-domain the N1 sub-domain, while the proximal region is called the N2 sub-domain. Note that

while the whole N-terminal region of IF2 is of variable length, the C-terminal region composed of the core domains (G, II, C1, and C2) is highly conserved between bacterial species. Sub-domain N1 is reportedly connected to IF2 (N2 sub-domain and core domains) through a flexible linker[29]. **b** Sequence alignment of the N-terminal region in *P. aeruginosa*, *E. coli*, and *T. thermophilus* IF2. The sub-domain regions for which structural information is available is underlined and color coded according to the bacterial species for which it belongs. The structure of the N2 sub-domain of *P. aeruginosa* IF2 is reported in this work. **c** Sequence alignment of the N1 and N2 sub-domains of IF2 in *E. coli* and *P. aeruginosa* with the N/G1 domain of *T. thermophilus* IF2, showing that the N1 and N2 sub-domains are sequence repeats. **d** Structure alignment of the solution structure of the N1 sub-domain from ribosome-free *E. coli* IF2 (PDB 1ND9[30]) with the N2 sub-domain of *P. aeruginosa* IF2-GDPCP reported here. The structures are highly similar with a root-mean-square-deviation (RMSD) value of 4.9 Å. **e** Structure alignment of the N/G1 domain from the crystal structure of ribosome-free *T. thermophilus* IF2 (PDB 4KJZ[31]) with the N2 sub-domain of *P. aeruginosa* IF2-GDPCP shows an almost perfect fit with RMSD of 1 Å.

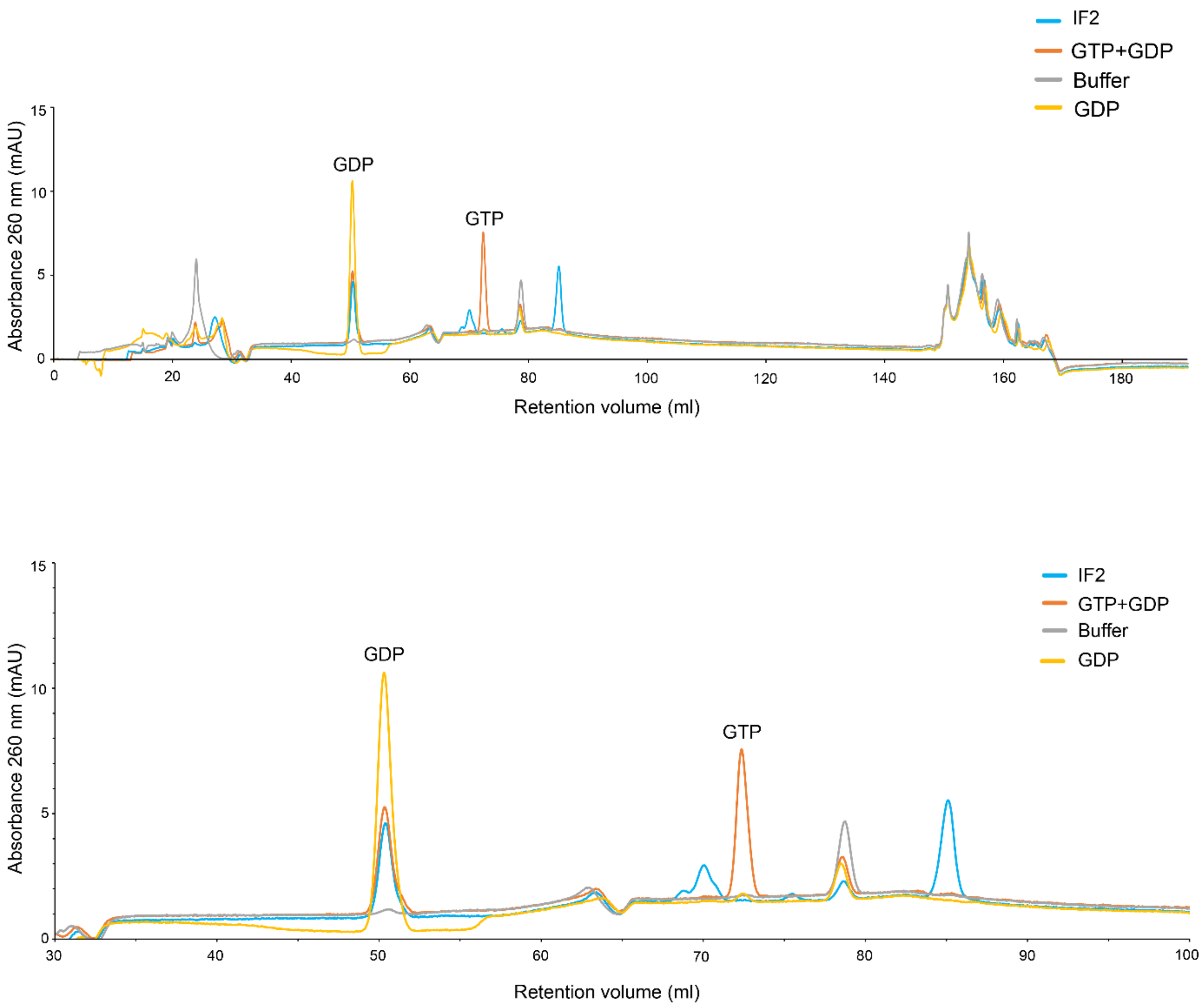

Supplementary Fig. 2. *Pseudomonas aeruginosa* IF2 co-purifies with GDP.
Reverse-phase HPLC analysis of GDP (yellow trace) and a mixture of GDP and GTP (orange trace). The gray trace is the control with buffer only. The blue trace is that of purified IF2 which shows the presence of GDP. Upper panel represents the full chromatogram and the lower panel focuses on the retention volume between 30 and 100 ml.

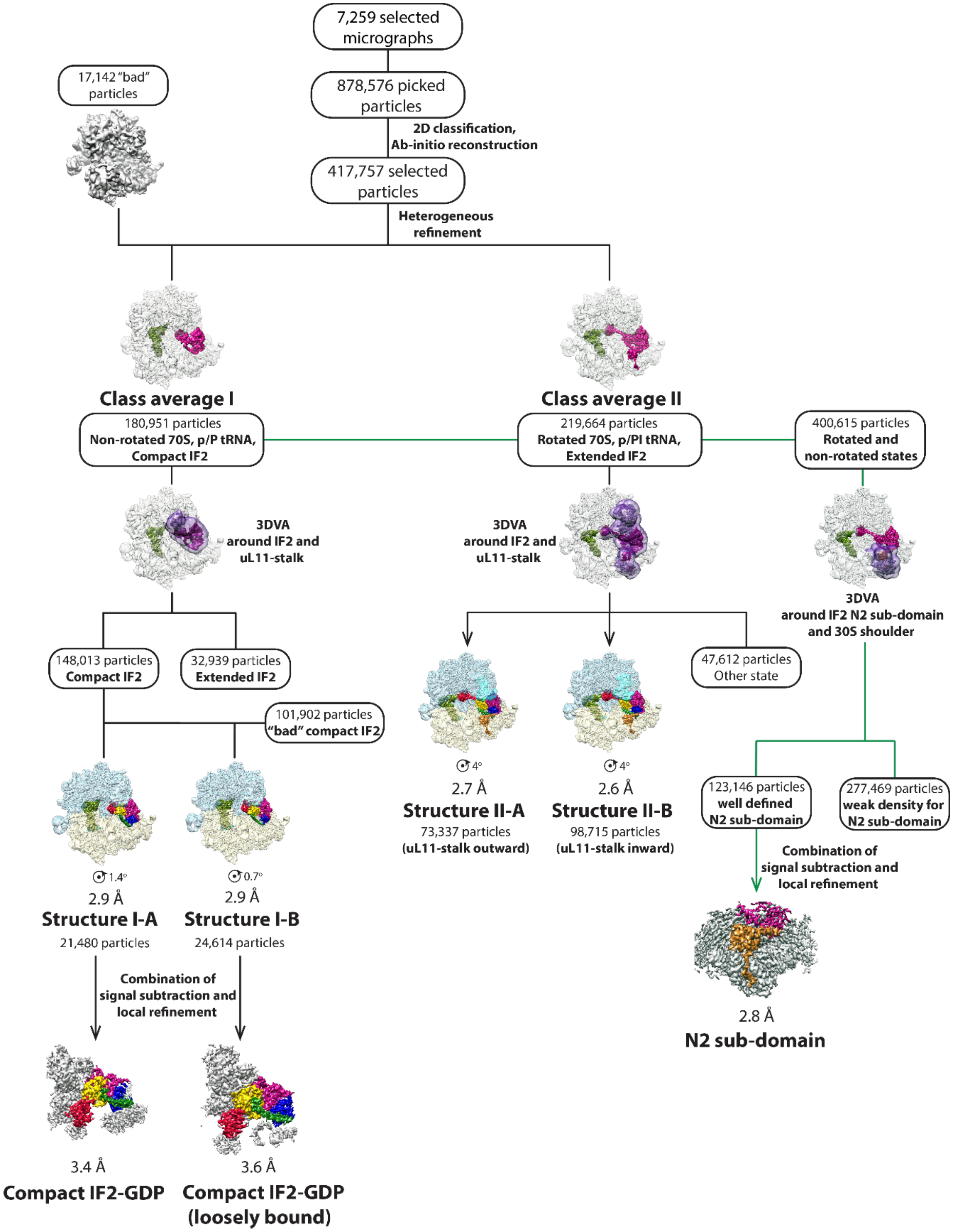

Supplementary Fig. 3. Cryo-EM data processing and particle classification workflow. Scheme of the data processing workflow. All steps were performed in cryoSPARC 3.1.0[32]. 8,056 micrographs were collected, of which 7,259 were selected for further processing. After two rounds of reference-free 2D classification, the selected particles were used to generate *ab-initio* volumes.

Particles from the 3D volumes that appeared to be the 70S ribosome based on their shape and size were separated according to their ratcheted state using heterogeneous refinement, yielding two main classes of particles (class averages I and II). Out of the non-ratcheted 180,951 particles (class average I), 148,013 were separated for containing density for compact IF2 using 3D variability analysis around IF2 and the uL11-stalk region. The process discarded 32,939 particles containing fragmented density for extended IF2. Similarly, we discarded 101,902 particles that had sparse density for compact IF2. The remaining particles belonged to two clusters with continuous density for compact IF2, which after refinement yielded structures I-A and I-B. Local refinement combined with particle subtraction of compact IF2 yielded the final maps of compact IF2-GDP. Out of the ratcheted 219,664 particles (class average II), 172,052 were separated for containing well-defined density for extended IF2 using 3D variability analysis around IF2 and the uL11-stalk region, as performed for the compact IF2. This process allowed sorting particles that differed only in the position of the uL11-stalk, which after refinement yielded structures II-A and II-B. The best EM map for the N2 sub-domain was obtained using particles from both class averages I and II (green lines). Out of the non-rotated (class average I) and rotated (class average II) 400,615 particles, 123,146 were separated using variability analysis around the N2 globular sub-domain for containing continuous density for this region of IF2. Subsequent combination of local refinement and particle subtraction resulted in the high-resolution reconstruction of the N2 sub-domain of IF2.

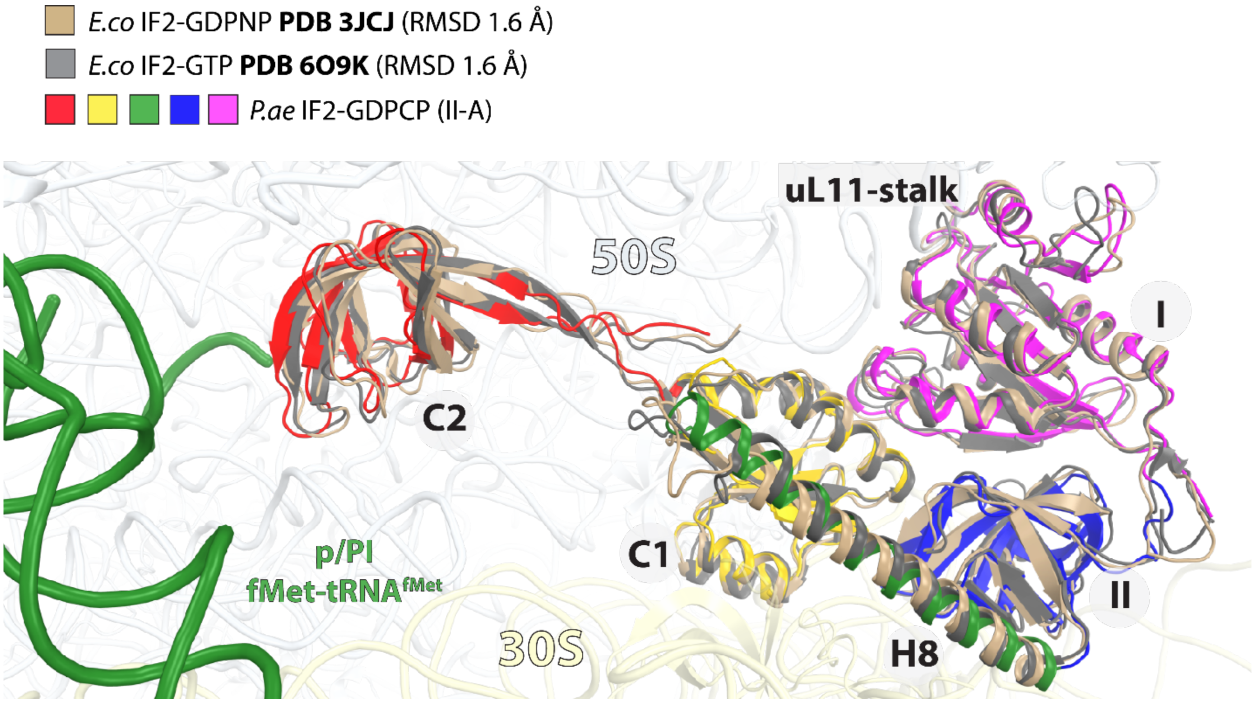

Supplementary Fig. 4. Structure alignment of the GTP-bound form of IF2 in the 70S-IC. IF2-GDPCP in the *P. aeruginosa* 70S-IC reported here adopts the same conformation as the previously reported IF2-GDPNP (PDB 3JCJ[33]) and IF2-GTP (PDB 6O9K[34]) in the *E. coli* 70S-IC, with superimpositions yielding RMSD values of 1.6 Å.

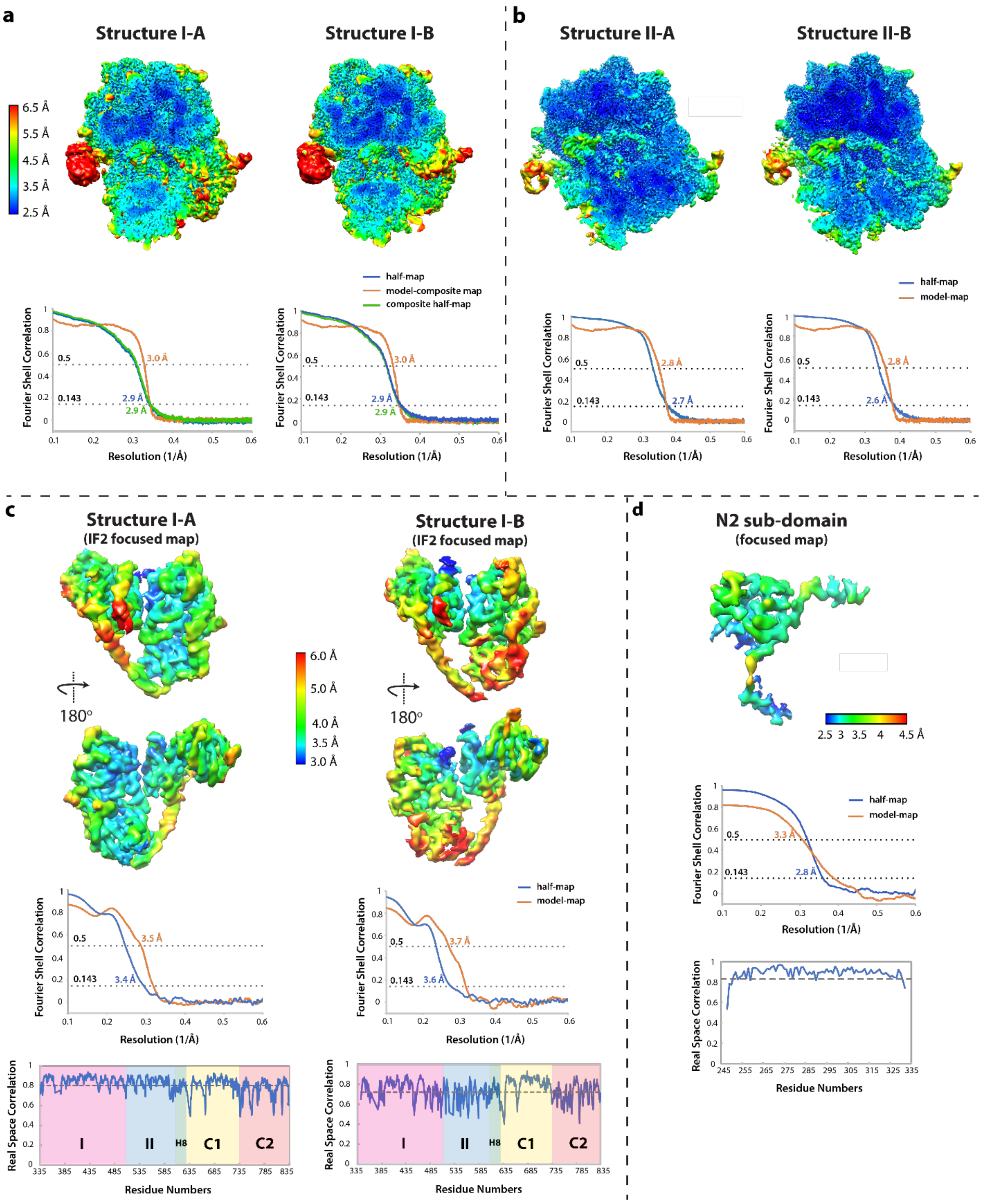

Supplementary Fig. 5. Local resolution estimation and Fourier Shell Correlation (FSC) validation. Local resolution heat maps on slices of density from (**a**) structures I-A, I-B and (**b**) structures II-A, II-B shown in the range of 2.5 Å – 6.5 Å resolution, calculated with cryoSPARC 3.1.0 implementation of BlocRes[35]. Below each reconstruction, gold-standard FSC curves of each

half-map (cyan), using a ‘soft mask’ excluding solvent (blue) and model-map (orange), are plotted across resolution. Map and model validation were performed in PHENIX 1.19.2[36]. For structures I-A and I-B, for which composite maps were generated from EM maps of IF2 and the 70S ribosome obtained after focused refinement, additional FSC curves for the composite EM map (green) and the model vs composite map fit (orange) are shown. Local resolution maps, FSC curves of half-maps (masked with a ‘soft mask’) and model-map FSC for (**c**) compact IF2 from structures I-A and I-B and (**d**) N2 sub-domain are shown. At the bottom of panels (**c**) and (**d**), the real space correlation coefficient (RSCC) plot across all residues of the model is shown. The mean RSCC value is plotted as a dotted line and color shaded areas delineate the respective domains in compact IF2.

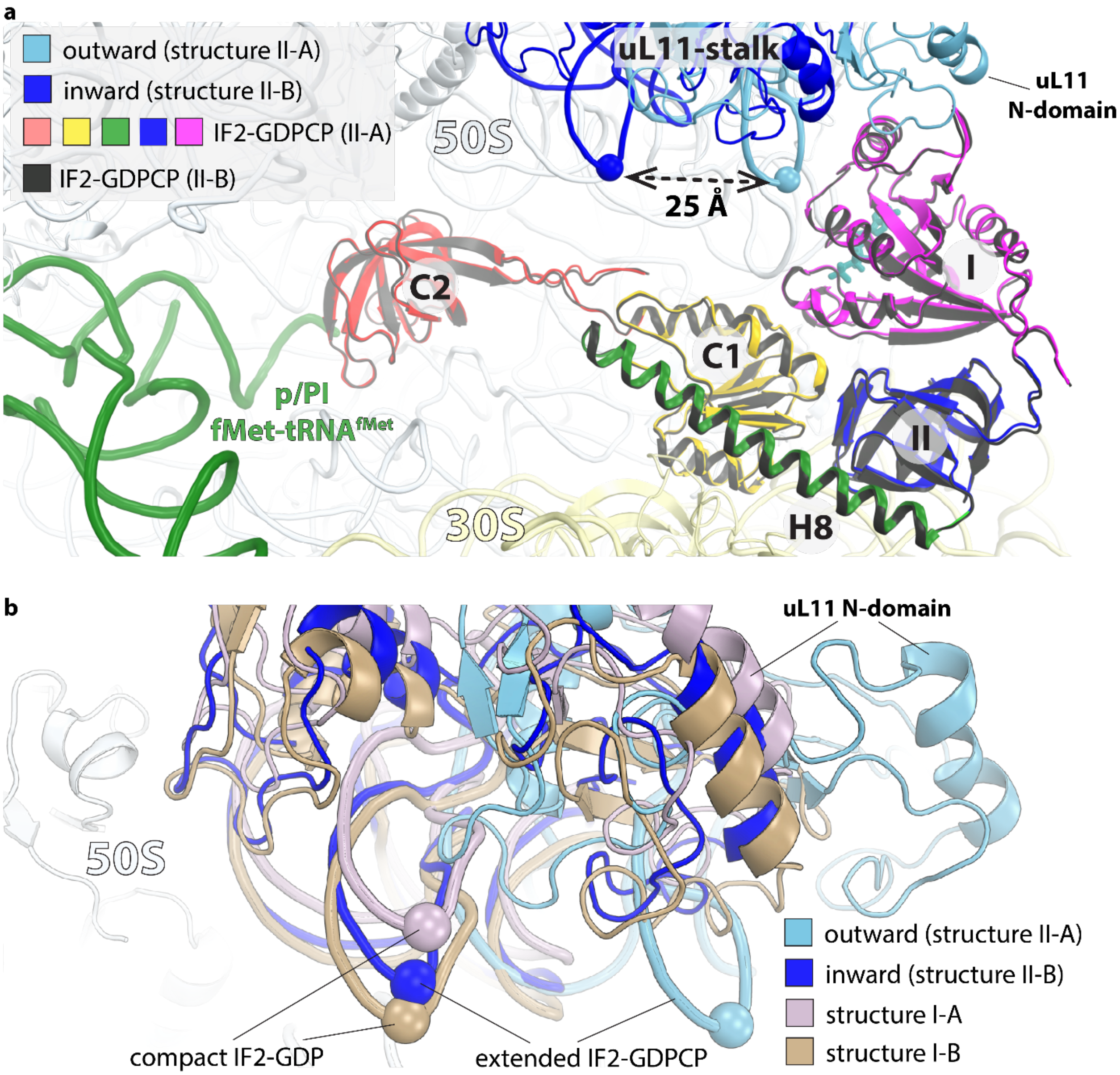

Supplementary Fig. 6. Movement of the uL11-stalk in the 70S-IC. a Alignment of the 23S rRNA in structures II-A and II-B with extended IF2-GDPCP reveals the movement of the uL11-stalk in the 70S-IC. The displacement of the uL11-stalk is measured between the phosphate (shown as spheres) of nucleotide A1057 (*E. coli* A1067) in helix H43. With the uL11-stalk in the outward position in structure II-A, the N-domain of protein uL11 interacts with the G-domain of IF2. Domains of extended IF2 in structure II-A are colored as in Figure 1d, while IF2 from structure II-B is black. **b** In structures I-A and I-B with compact IF2-GDP, the uL11-stalk locates in the inward position similar to that in structure II-B.

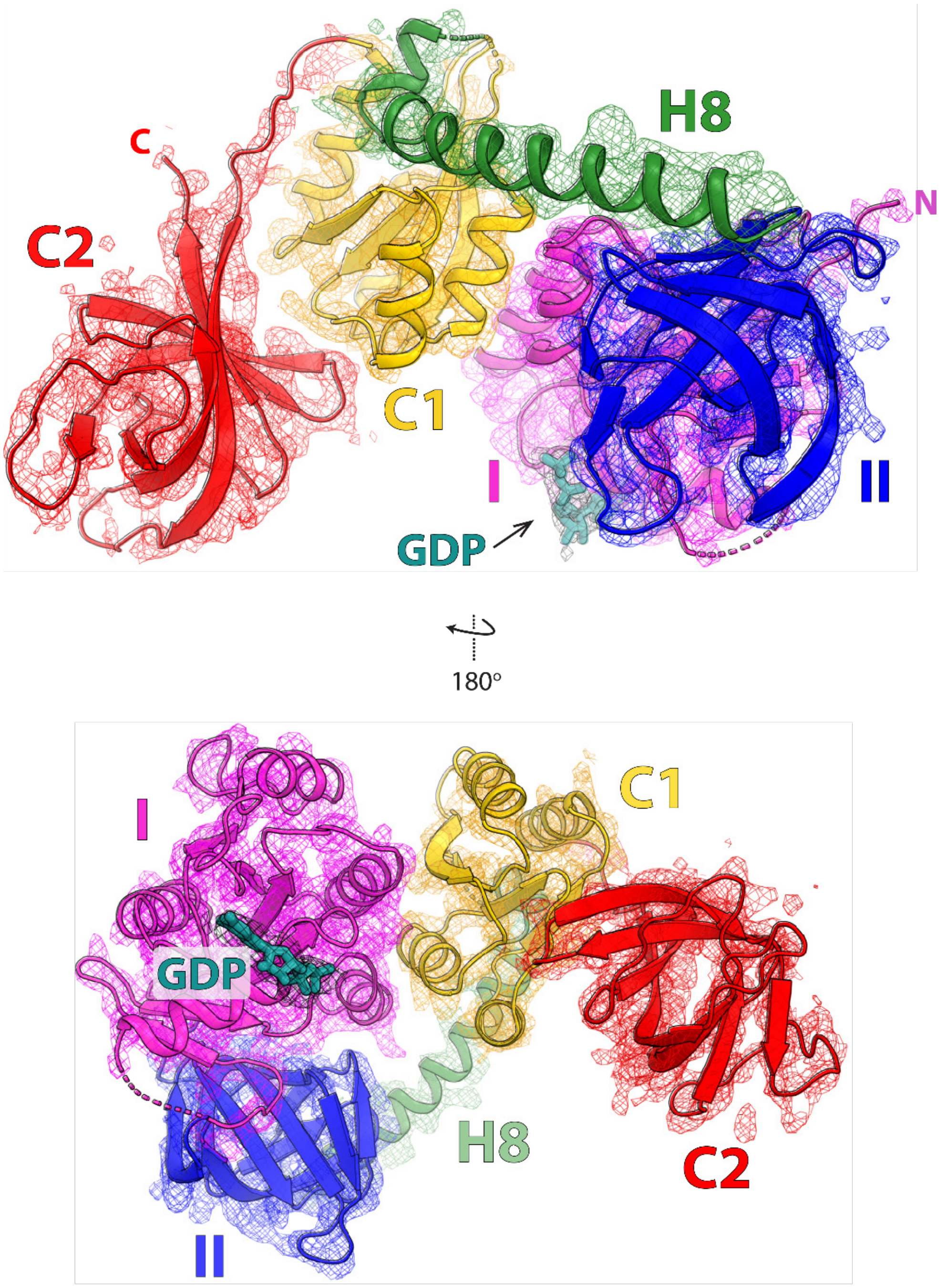

Supplementary Fig. 7. Cryo-EM density of compact IF2-GDP from structure I-A obtained after focused refinement. Domains of IF2 are colored as in Figure 1: G-domain (I, magenta), domain II (blue), helix H8 (green), domain C1 (yellow), domain C2 (red). The GDP nucleotide is colored teal with black mesh density contoured at 7σ. Experimental EM density shown as colored mesh contoured at 8σ for domains I, II and C1, 6σ for domain C2, and 5.5σ for helix H8.

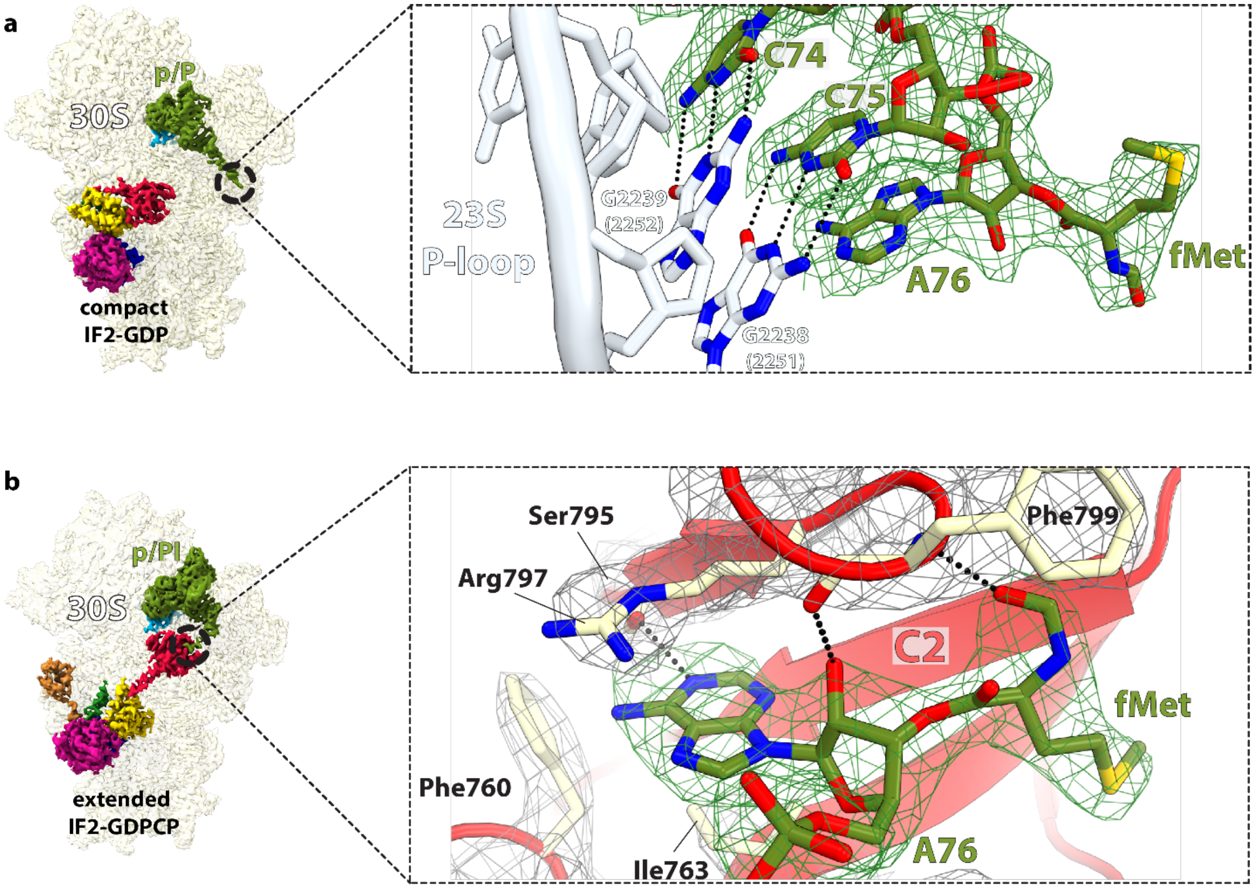

Supplementary Fig. 8. Interactions of initiator tRNA with the ribosome in structure I-A and with domain C2 of IF2 in structure II-A. a In structure I-A, initiator fMet-tRNA$_i^{fMet}$ is fully accommodated in the P site of the 70S-IC. The CCA-end of tRNA participates in canonical Watson-Crick base pairing interactions with the 23S P-loop, positioning the fMet moiety in the peptidyl transferase center (PTC) as previously reported[37-39]. The EM density for the 3'CCA and fMet is shown with green mesh. **b** In structure II-A, domain C2 (red) in extended IF2 interacts with A76-fMet (green) of initiator tRNA bound in the p/PI state. The EM density mesh for A76-fMet (green) and key IF2 residues (gray) are shown. Domain C2 of *P. aeruginosa* IF2 interacts with the 3'CCA and the fMet moiety of initiator tRNA as reported in the *E. coli* 70S-IC[33,34].

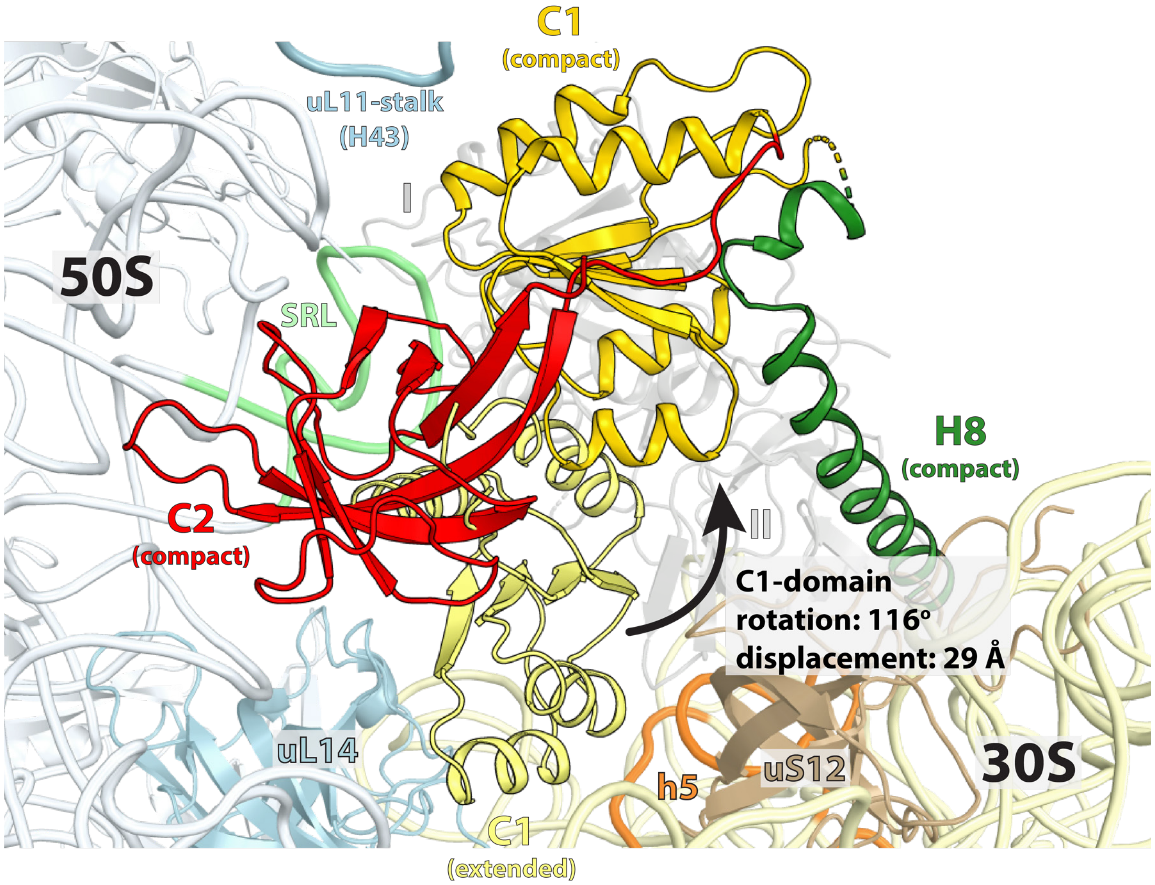

Supplementary Fig. 9. Position of domain C1 in extended IF2 (pale yellow) relative to its new location in compact IF2 (yellow). In extended IF2-GDPCP (structures II-A and II-B), domain C1 (pale yellow) localizes in the cleft between the 30S and 50S subunits (see Supplementary Fig. 11a, b). Alignment of domains I and II (gray) of extended (II-A) and compact (I-A) IF2 reveals the rotation and displacement of domain C1 to its new position (yellow) under the uL11-stalk.

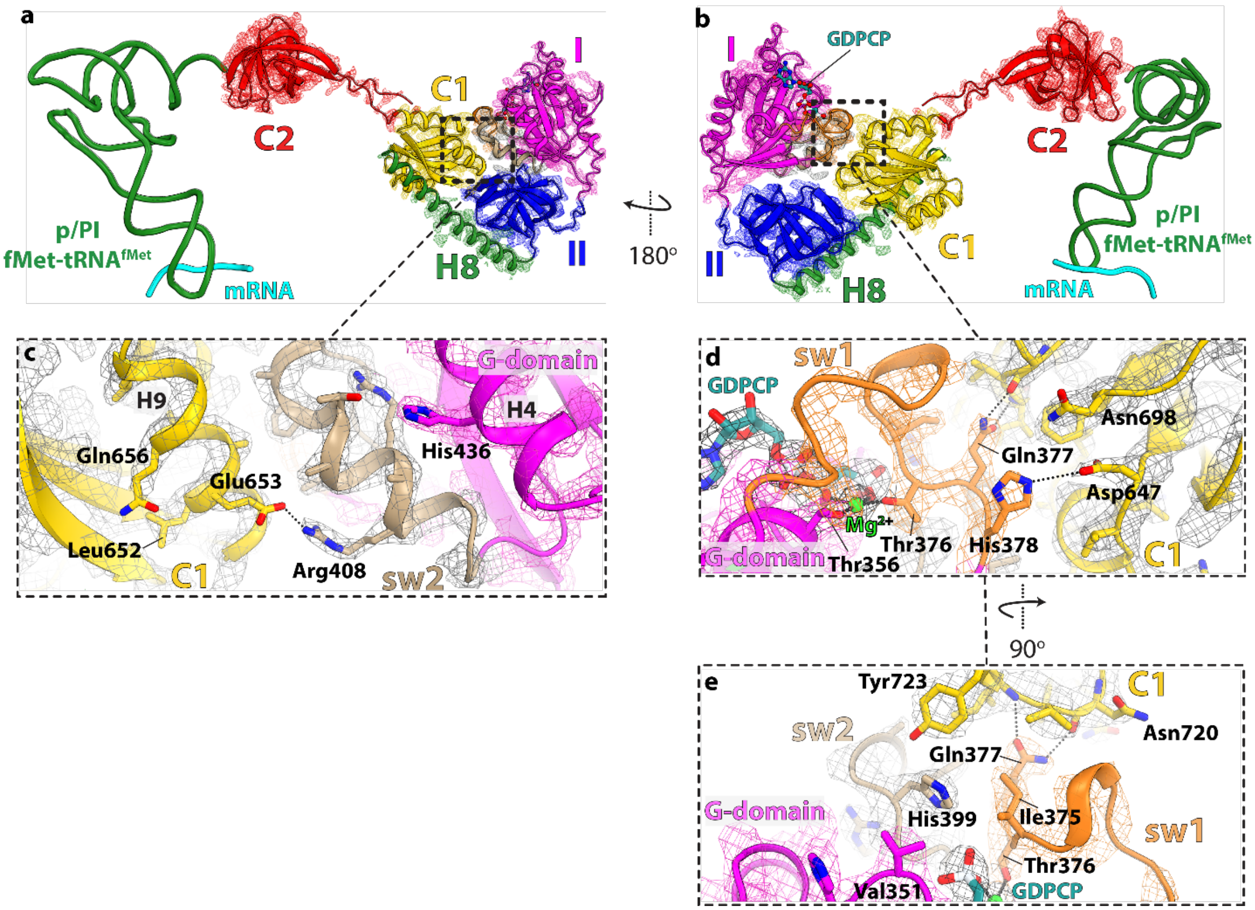

Supplementary Fig. 10. Interactions between the G- and C1-domains in extended IF2 in the 70S-IC. a, b Overview of extended IF2-GDPCP bound to the 70S ribosome in structure II-A. **c** Magnified view of the interactions between the sw2-helix in the G-domain and helix H9 in domain C1. **d, e** Two magnified views of the interactions between the sw1 loop and domain C1. In extended IF2, sw1 and sw2 contribute to the structural rigidity of domain C1 located in a cleft formed between the 30S and 50S subunits (see Supplementary Fig. 11a, b). The EM density is shown with colored mesh. Domains and the switch regions in IF2 are colored as in the main text figures.

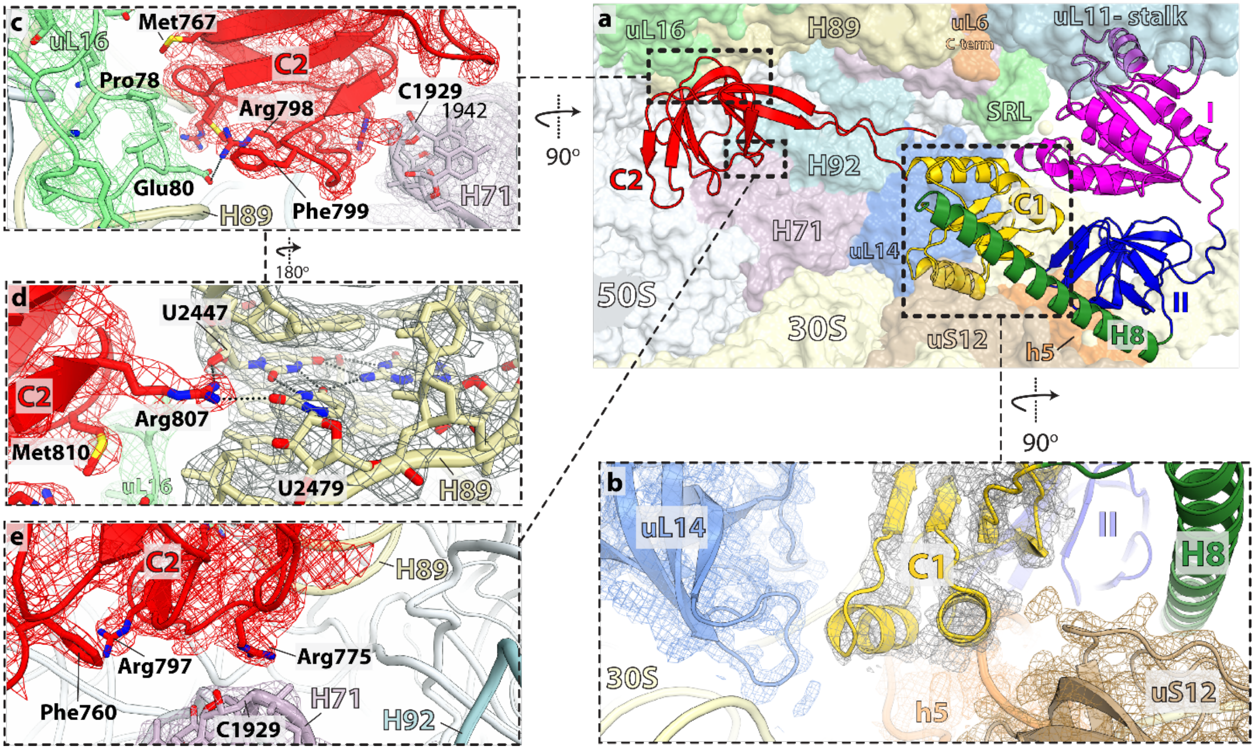

Supplementary Fig. 11. Interactions between the C1- and C2-domains of extended IF2 and the ribosome in the 70S-IC. a Overview of extended IF2-GDPCP bound to the 70S ribosome in structure II-A, with ribosomal elements and helices labeled. **b** Magnified view showing the location of domain C1 in the cleft formed by ribosomal protein uL14, 16S rRNA helix h5, and protein uS12. **c, d** Magnified views of the interactions between domain C2 and the 50S subunit. **c** In extended IF2, domain C2 interacts with ribosomal protein uL16, with residues Arg^{798} of IF2 and Glu^{80} of uL16 located within hydrogen bonding distance. **d** Residue Arg^{807} of IF2 is involved in a network of hydrogen bonds with base pair formed between nucleotides U2447 (*E. coli* 2460) and U2479 (2492) located at the base of helix H89 in 23S rRNA. **e** Residue Arg^{775} forms a stacking interaction with the ribose of nucleotide C1929 (1942) in H71 of 23S rRNA. The EM density is shown with colored mesh. Domains and the switch regions in IF2 are colored as in the main text figures.

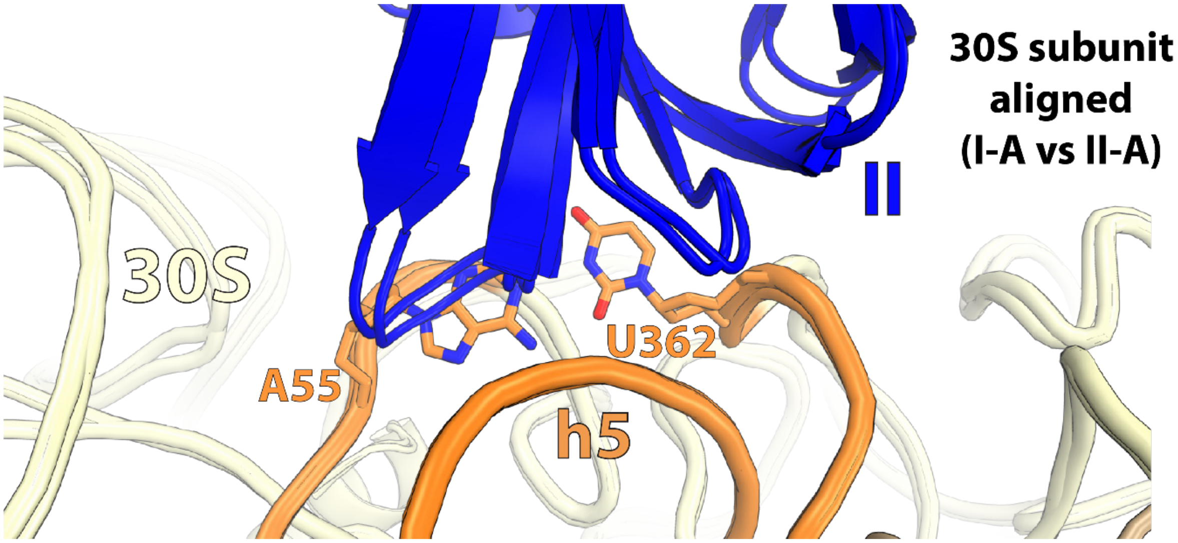

Supplementary Fig. 12. Position of domain II in compact and extended IF2. Superimposition of the 30S subunit (yellow) in structures I-A and II-A shows that domain II (blue) is identically positioned in extended and compact IF2, stabilized by stacking interactions with base pair A55-U362 (*E. coli* A55-U368). The view is the same as in main text Figure 5d.

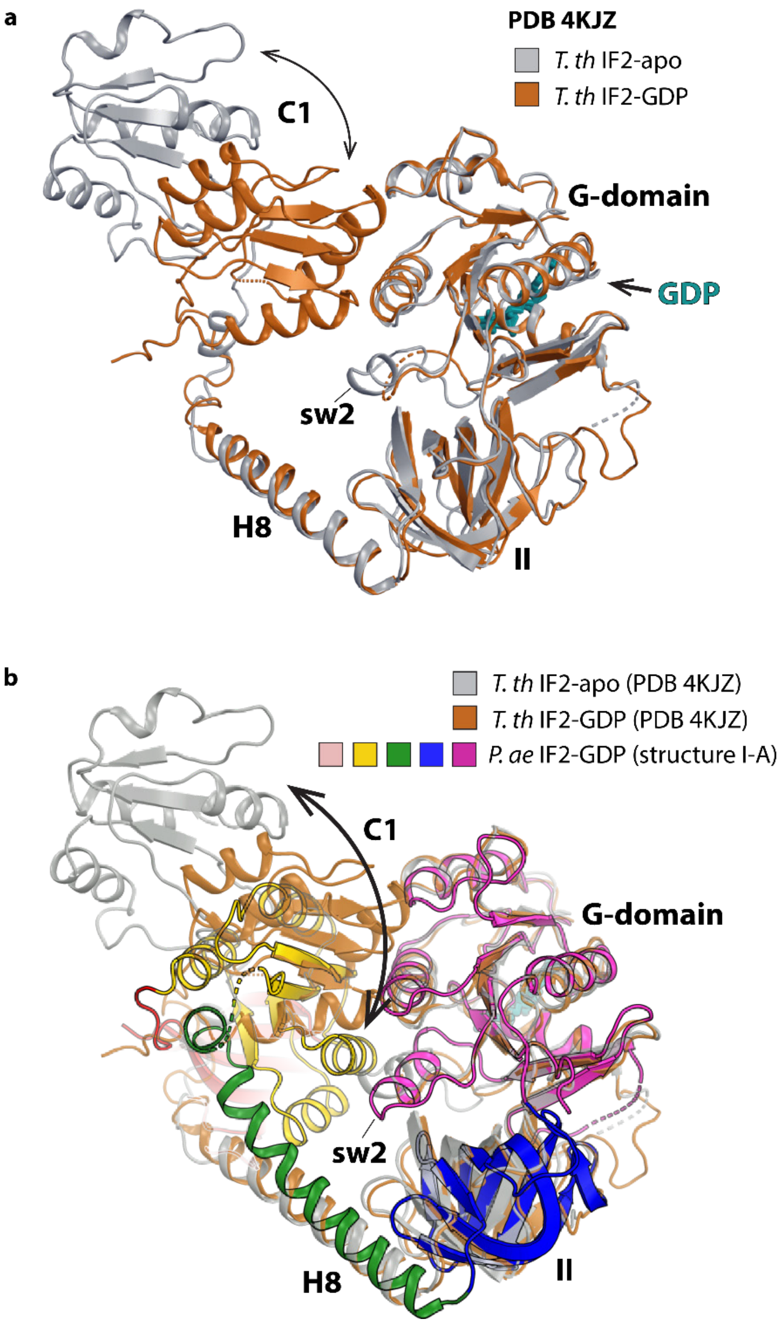

Supplementary Fig. 13. Comparison of compact IF2-GDP (structure I-A) with the crystal structures of *Thermus thermophilus* apo-IF2 and IF2-GDP [PDB 4KJZ[31]]. a The superimposition of the *T. thermophilus* apo-IF2 (gray) and IF2-GDP (brown) crystal structures shows that domain C1 is flexibly disposed relative to domains G, II and helix H8. **b** Alignment of the G-domain of compact IF2-GDP from structure I-A (colored domains) with that of *T. thermophilus* apo-IF2 (gray) and IF2-GDP (brown) shows that domain C1 is flexible and has a wide range of motion. The sw2 region in the crystal structure of *T. thermophilus* IF2-GDP is disordered, and in *T. thermophilus* apo-IF2 it is oriented similar to that in extended IF2-GDPCP from structures II-A and II-B (not shown).

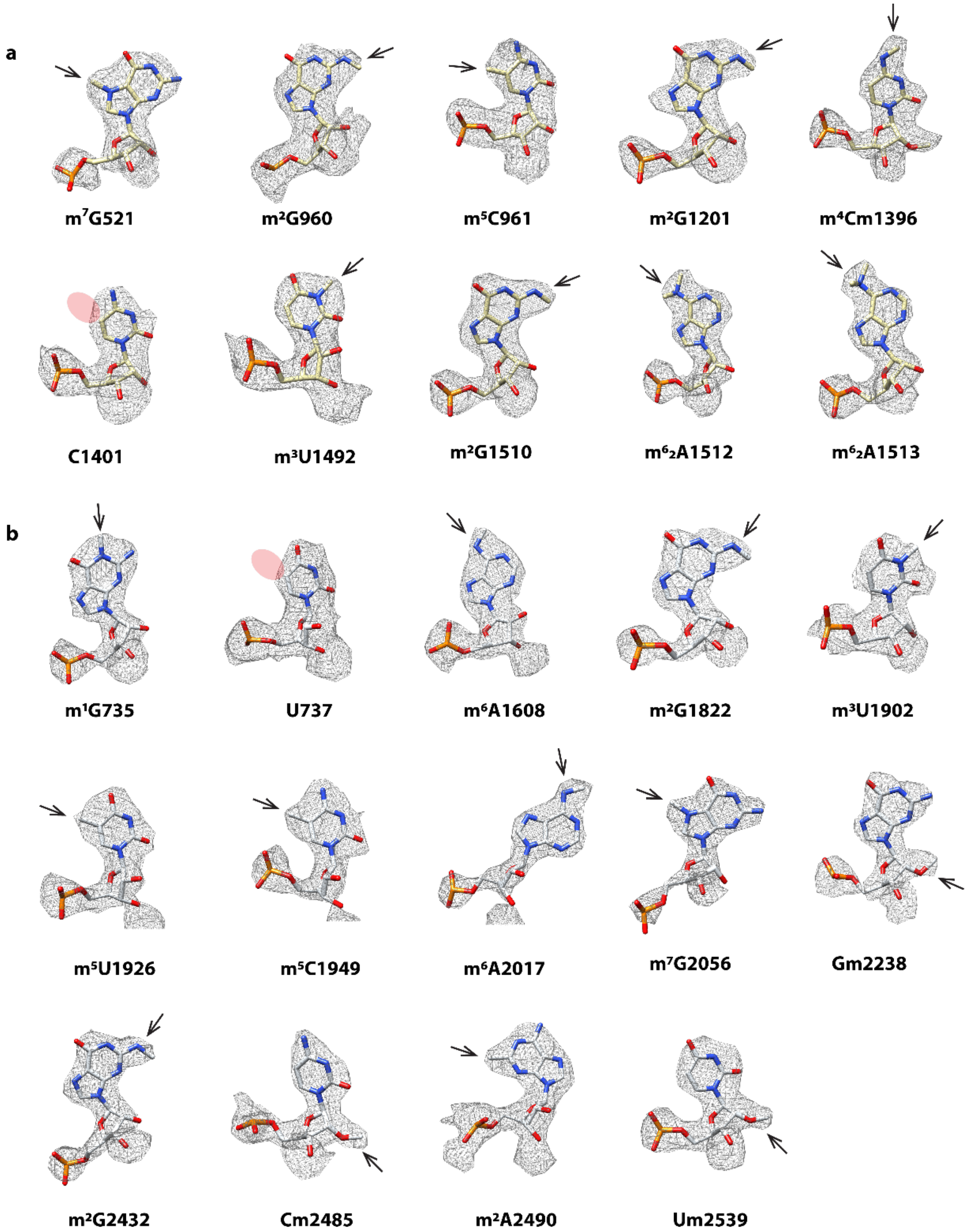

Supplementary Fig. 14. EM density of methylated nucleotides modeled into the 16S (a) and 23S (b) rRNAs of structures I-A, I-B, II-A, and II-B. The arrow next to each nucleotide indicates the modification. Pink circles next to nucleotides C1401 in the 16S rRNA and U737 in the 23S rRNA indicate position C5 where extra density for a methyl group was expected based on the high-resolution cryo-EM structure of the *E. coli* 70S ribosome[40]. The missing densities is explained by the absence of the rRNA modification enzymes in *P. aeruginosa* PAO1 that methylate these nucleotides in *E. coli* (see Supplementary Table 2). The *P. aeruginosa* nucleotide numbering is used and the corresponding *E. coli* numbers are provided in Supplementary Table 2.

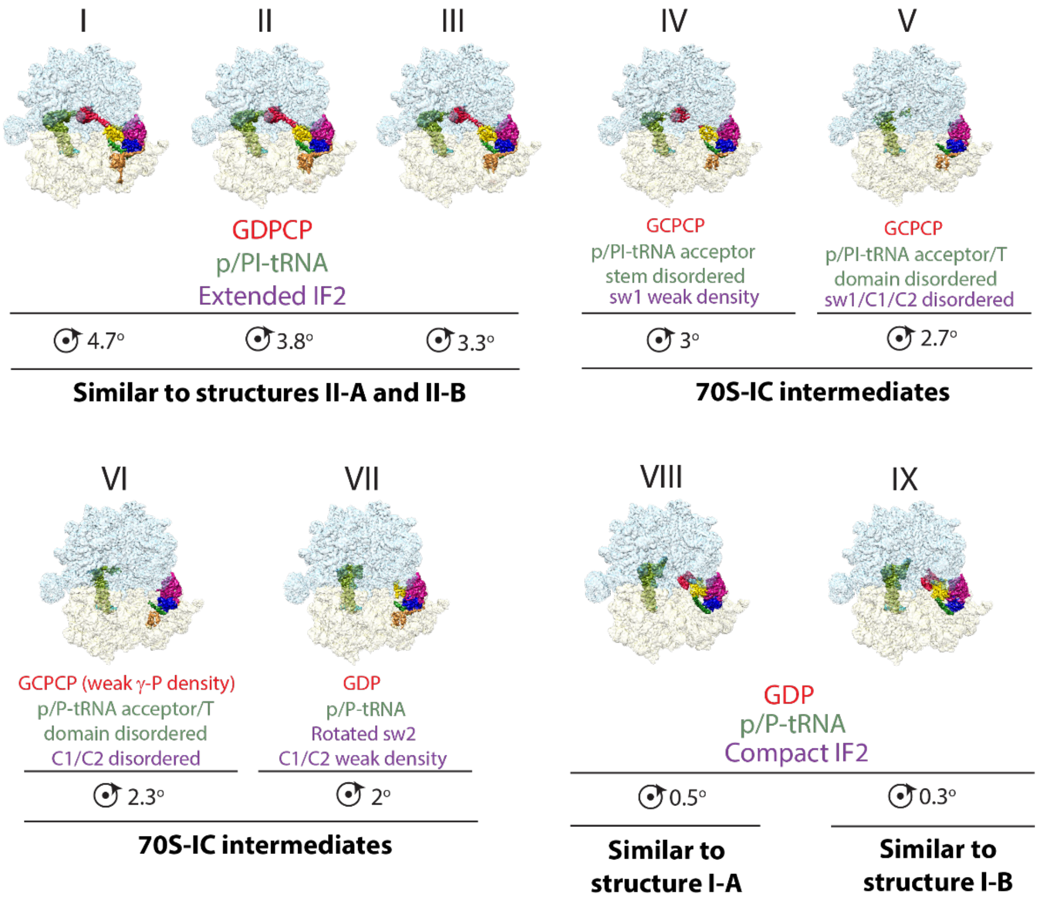

Supplementary Fig. 15. Nine 3D reconstructions of the 70S-IC based on global 3D variability analysis (see Supplementary Discussion). Class averages I to IX were grouped based on the extent of 30S subunit rotation relative to the non-rotated *P. aeruginosa* 70S ribosome [PDB 6SPG[41]]. The degree of 30S subunit rotation, the G-nucleotide bound, the state of fMet-tRNA$_i^{fMet}$ binding, and features of IF2 are indicated under each class average. The 50S and 30S subunits are shown in light blue and yellow, respectively. The initiator tRNA is green and domains of IF2 are colored as in Figure 1. The volumes of the nine class averages were morphed together to generate Supplementary Movie 2.

Supplementary References

1. Okamoto, S. et al. Loss of a conserved 7-methylguanosine modification in 16S rRNA confers low-level streptomycin resistance in bacteria. *Mol. Microbiol.* **63**, 1096-106 (2007).
2. Lesnyak, D.V. et al. Methyltransferase that modifies guanine 966 of the 16 S rRNA: functional identification and tertiary structure. *J. Biol. Chem.* **282**, 5880-7 (2007).
3. Gu, X.R., Gustafsson, C., Ku, J., Yu, M. & Santi, D.V. Identification of the 16S rRNA m5C967 methyltransferase from Escherichia coli. *Biochemistry* **38**, 4053-7 (1999).
4. Tscherne, J.S. et al. Purification, cloning, and characterization of the 16S RNA m5C967 methyltransferase from Escherichia coli. *Biochemistry* **38**, 1884-92 (1999).
5. Tscherne, J.S., Nurse, K., Popienick, P. & Ofengand, J. Purification, cloning, and characterization of the 16 S RNA m2G1207 methyltransferase from Escherichia coli. *J. Biol. Chem.* **274**, 924-9 (1999).
6. Carrion, M., Gomez, M.J., Merchante-Schubert, R., Dongarra, S. & Ayala, J.A. mraW, an essential gene at the dcw cluster of Escherichia coli codes for a cytoplasmic protein with methyltransferase activity. *Biochimie* **81**, 879-88 (1999).
7. Kimura, S. & Suzuki, T. Fine-tuning of the ribosomal decoding center by conserved methyl-modifications in the Escherichia coli 16S rRNA. *Nucl. Acids Res.* **38**, 1341-52 (2010).
8. Gutierrez, B., Douthwaite, S. & Gonzalez-Zorn, B. Indigenous and acquired modifications in the aminoglycoside binding sites of Pseudomonas aeruginosa rRNAs. *RNA Biol.* **10**, 1324-32 (2013).
9. Andersen, N.M. & Douthwaite, S. YebU is a m5C methyltransferase specific for 16 S rRNA nucleotide 1407. *J. Mol. Biol.* **359**, 777-86 (2006).
10. Basturea, G.N., Rudd, K.E. & Deutscher, M.P. Identification and characterization of RsmE, the founding member of a new RNA base methyltransferase family. *RNA* **12**, 426-34 (2006).
11. Basturea, G.N., Dague, D.R., Deutscher, M.P. & Rudd, K.E. YhiQ is RsmJ, the methyltransferase responsible for methylation of G1516 in 16S rRNA of E. coli. *J. Mol. Biol.* **415**, 16-21 (2012).
12. Helser, T.L., Davies, J.E. & Dahlberg, J.E. Mechanism of kasugamycin resistance in Escherichia coli. *Nat. New Biol.* **235**, 6-9 (1972).
13. Poldermans, B., Roza, L. & Van Knippenberg, P.H. Studies on the function of two adjacent N6,N6-dimethyladenosines near the 3' end of 16 S ribosomal RNA of Escherichia coli. III. Purification and properties of the methylating enzyme and methylase-30 S interactions. *J. Biol. Chem.* **254**, 9094-100 (1979).
14. Gustafsson, C. & Persson, B.C. Identification of the rrmA gene encoding the 23S rRNA m1G745 methyltransferase in Escherichia coli and characterization of an m1G745-deficient mutant. *J. Bacteriol.* **180**, 359-65 (1998).
15. Madsen, C.T., Mengel-Jorgensen, J., Kirpekar, F. & Douthwaite, S. Identifying the methyltransferases for m(5)U747 and m(5)U1939 in 23S rRNA using MALDI mass spectrometry. *Nucl. Acids Res.* **31**, 4738-46 (2003).
16. Sergiev, P.V., Serebryakova, M.V., Bogdanov, A.A. & Dontsova, O.A. The ybiN gene of Escherichia coli encodes adenine-N6 methyltransferase specific for modification of A1618 of 23 S ribosomal RNA, a methylated residue located close to the ribosomal exit tunnel. *J. Mol. Biol.* **375**, 291-300 (2008).
17. Sergiev, P.V., Lesnyak, D.V., Bogdanov, A.A. & Dontsova, O.A. Identification of Escherichia coli m2G methyltransferases: II. The ygjO gene encodes a methyltransferase specific for G1835 of the 23 S rRNA. *J. Mol. Biol.* **364**, 26-31 (2006).
18. Ero, R., Peil, L., Liiv, A. & Remme, J. Identification of pseudouridine methyltransferase in Escherichia coli. *RNA* **14**, 2223-33 (2008).
19. Purta, E., Kaminska, K.H., Kasprzak, J.M., Bujnicki, J.M. & Douthwaite, S. YbeA is the m3Psi methyltransferase RlmH that targets nucleotide 1915 in 23S rRNA. *RNA* **14**, 2234-44 (2008).
20. Agarwalla, S., Kealey, J.T., Santi, D.V. & Stroud, R.M. Characterization of the 23 S ribosomal RNA m5U1939 methyltransferase from Escherichia coli. *J. Biol. Chem.* **277**, 8835-40 (2002).
21. Purta, E., O'Connor, M., Bujnicki, J.M. & Douthwaite, S. YccW is the m5C methyltransferase specific for 23S rRNA nucleotide 1962. *J. Mol. Biol.* **383**, 641-51 (2008).
22. Golovina, A.Y. et al. The last rRNA methyltransferase of E. coli revealed: the yhiR gene encodes adenine-N6 methyltransferase specific for modification of A2030 of 23S ribosomal RNA. *RNA* **18**, 1725-34 (2012).
23. Kimura, S. et al. Base methylations in the double-stranded RNA by a fused methyltransferase bearing unwinding activity. *Nucl. Acids Res.* **40**, 4071-85 (2012).

24. Lovgren, J.M. & Wikstrom, P.M. The rlmB gene is essential for formation of Gm2251 in 23S rRNA but not for ribosome maturation in Escherichia coli. *J. Bacteriol.* **183**, 6957-60 (2001).
25. Purta, E., O'Connor, M., Bujnicki, J.M. & Douthwaite, S. YgdE is the 2'-O-ribose methyltransferase RlmM specific for nucleotide C2498 in bacterial 23S rRNA. *Mol. Microbiol.* **72**, 1147-58 (2009).
26. Toh, S.M., Xiong, L., Bae, T. & Mankin, A.S. The methyltransferase YfgB/RlmN is responsible for modification of adenosine 2503 in 23S rRNA. *RNA* **14**, 98-106 (2008).
27. Bugl, H. et al. RNA methylation under heat shock control. *Mol. Cell* **6**, 349-60 (2000).
28. Caldas, T. et al. The FtsJ/RrmJ heat shock protein of Escherichia coli is a 23 S ribosomal RNA methyltransferase. *J. Biol. Chem.* **275**, 16414-9 (2000).
29. Laursen, B.S., Kjaergaard, A.C., Mortensen, K.K., Hoffman, D.W. & Sperling-Petersen, H.U. The N-terminal domain (IF2N) of bacterial translation initiation factor IF2 is connected to the conserved C-terminal domains by a flexible linker. *Protein Sci.* **13**, 230-9 (2004).
30. Laursen, B.S., Mortensen, K.K., Sperling-Petersen, H.U. & Hoffman, D.W. A conserved structural motif at the N terminus of bacterial translation initiation factor IF2. *J. Biol. Chem.* **278**, 16320-8 (2003).
31. Eiler, D., Lin, J., Simonetti, A., Klaholz, B.P. & Steitz, T.A. Initiation factor 2 crystal structure reveals a different domain organization from eukaryotic initiation factor 5B and mechanism among translational GTPases. *Proc. Natl. Acad. Sci. USA* **110**, 15662-7 (2013).
32. Punjani, A., Rubinstein, J.L., Fleet, D.J. & Brubaker, M.A. cryoSPARC: algorithms for rapid unsupervised cryo-EM structure determination. *Nat. Methods* **14**, 290-296 (2017).
33. Sprink, T. et al. Structures of ribosome-bound initiation factor 2 reveal the mechanism of subunit association. *Sci. Adv.* **2**, e1501502 (2016).
34. Kaledhonkar, S. et al. Late steps in bacterial translation initiation visualized using time-resolved cryo-EM. *Nature* **570**, 400-404 (2019).
35. Cardone, G., Heymann, J.B. & Steven, A.C. One number does not fit all: mapping local variations in resolution in cryo-EM reconstructions. *J. Struct. Biol.* **184**, 226-36 (2013).
36. Afonine, P.V. et al. New tools for the analysis and validation of cryo-EM maps and atomic models. *Acta Crystallogr. D Struct. Biol.* **74**, 814-840 (2018).
37. Polikanov, Y.S., Steitz, T.A. & Innis, C.A. A proton wire to couple aminoacyl-tRNA accommodation and peptide-bond formation on the ribosome. *Nat. Struct. Mol. Biol.* **21**, 787-93 (2014).
38. Jin, H., Kelley, A.C., Loakes, D. & Ramakrishnan, V. Structure of the 70S ribosome bound to release factor 2 and a substrate analog provides insights into catalysis of peptide release. *Proc. Natl. Acad. Sci. USA* **107**, 8593-8 (2010).
39. Lin, J., Gagnon, M.G., Bulkley, D. & Steitz, T.A. Conformational Changes of Elongation Factor G on the Ribosome during tRNA Translocation. *Cell* **160**, 219-27 (2015).
40. Watson, Z.L. et al. Structure of the bacterial ribosome at 2 A resolution. *Elife* **9**(2020).
41. Halfon, Y. et al. Structure of Pseudomonas aeruginosa ribosomes from an aminoglycoside-resistant clinical isolate. *Proc. Natl. Acad. Sci. USA* **116**, 22275-22281 (2019).
