# Supplementary material for: Compact IF2 allows initiator tRNA accommodation into the P site and gates the ribosome to elongation
Source: Nat Commun. 2022 Jun 13;13:3388. doi: 10.1038/s41467-022-31129-2 (PMC9192638; doi:10.1038/s41467-022-31129-2)
Supplement: Supplementary file 3 — Description of Additional Supplementary Files [file 41467_2022_31129_MOESM3_ESM.pdf]

File name: Supplementary Movie 1

Description: Animation of the structural transitions in IF2, the ribosome, and initiator tRNA between structures I-A and II-A. The initial and last models are structures IIA and I-A, respectively, with the transitions rendered by morphing. The subunits of the ribosome, fMettRNA$_i^{fMet}$, mRNA, and domains of IF2 are colored as in Figure 1. As the 30S subunit rotates back in the 70S-IC, IF2 transitions from the extended to the compact conformation. The fMettRNA$_i^{fMet}$-binding domain C2 (red) collapses onto domain C1 (yellow) in compact IF2. The large-scale domain rearrangements in IF2 are concomitant with the movement of fMet-tRNA$_i^{fMet}$ from the p/PI state to the fully accommodated position into the P site. Domain C1 relocates under the uL11- stalk, 29 Å away from its location in extended IF2 on the rotated ribosome. The formation of the new interface between the G- and C1-domains is facilitated by the 65°-rotation of the switch 2 (sw2) α-helix region. The rearrangement of sw2 is dependent on the nucleotide-bound state of the Gdomain in IF2. The catalytic His^{399}, which in the GDPCP-bound IF2 interacts with the phosphate oxygen of A2649 (*E. coli* A2662) of the sarcinricin loop (SRL), is directed away from the nucleotide binding pocket in IF2- GDP.

File name: Supplementary Movie 2

Description: Animation of the 3D volume reconstructions from the global variability analysis performed using non-rotated (class average I) and rotated (class average II) 70S-IC ribosome particles (see Supplementary Discussion and Supplementary Fig. 15). The volume maps of the nine reconstructions shown in Supplementary Figure 15 are displayed sequentially as a continuous movie. The color scheme used for the ribosome, fMet-tRNA$_i^{fMet}$, mRNA, and IF2 is the same as that in Figure 1. On the rotated ribosome, IF2 is extended and its distal domain C2 (red) interacts with the 3'-end of fMet-tRNA$_i^{fMet}$ (green). Back-rotation of the 30S subunit and accommodation of fMettRNA$_i^{fMet}$ into the P site appear to correlate with the transition of IF2 to the compact conformation. During the transition from extended to compact IF2, domain C2 (red) collapses onto domain C1 (yellow) releasing fMettRNA$_i^{fMet}$ to the ribosome. Note that the EM density attributable to the N2 sub-domain in extended IF2 (brown) on the rotated ribosome is not visible in the non-rotated 70S-IC.
