# Supplementary material for: Compact IF2 allows initiator tRNA accommodation into the P site and gates the ribosome to elongation
Source: Nat Commun. 2022 Jun 13;13:3388. doi: 10.1038/s41467-022-31129-2 (PMC9192638; doi:10.1038/s41467-022-31129-2)
Supplement: Supplementary file 6 — Reporting Summary [file 41467_2022_31129_MOESM6_ESM.pdf]

nature portfolio

Corresponding author(s): Matthieu G. Gagnon

Last updated by author(s): May 23, 2022

Reporting Summary

Nature Portfolio wishes to improve the reproducibility of the work that we publish. This form provides structure for consistency and transparency in reporting. For further information on Nature Portfolio policies, see our Editorial Policies and the Editorial Policy Checklist.

Statistics

For all statistical analyses, confirm that the following items are present in the figure legend, table legend, main text, or Methods section.

n/a	Confirmed	
☒	☐	The exact sample size (*n*) for each experimental group/condition, given as a discrete number and unit of measurement
☒	☐	A statement on whether measurements were taken from distinct samples or whether the same sample was measured repeatedly
☒	☐	The statistical test(s) used AND whether they are one- or two-sided<br>*Only common tests should be described solely by name; describe more complex techniques in the Methods section.*
☒	☐	A description of all covariates tested
☒	☐	A description of any assumptions or corrections, such as tests of normality and adjustment for multiple comparisons
☐	☒	A full description of the statistical parameters including central tendency (e.g. means) or other basic estimates (e.g. regression coefficient) AND variation (e.g. standard deviation) or associated estimates of uncertainty (e.g. confidence intervals)
☒	☐	For null hypothesis testing, the test statistic (e.g. *F*, *t*, *r*) with confidence intervals, effect sizes, degrees of freedom and *P* value noted<br>*Give P values as exact values whenever suitable.*
☒	☐	For Bayesian analysis, information on the choice of priors and Markov chain Monte Carlo settings
☒	☐	For hierarchical and complex designs, identification of the appropriate level for tests and full reporting of outcomes
☒	☐	Estimates of effect sizes (e.g. Cohen's *d*, Pearson's *r*), indicating how they were calculated

Our web collection on statistics for biologists contains articles on many of the points above.

Software and code

Policy information about availability of computer code

Data collection	EPU (v. 2.11.1)
Data analysis	CryoSparc (v. 3.1.0), Phenix and Molprobity (v. 1.19.2-4158), Coot 0.8.9.1, PyMOL (v. 2.1.0), Chimera (v. 1.14), Chimera X (v. 1.12)

For manuscripts utilizing custom algorithms or software that are central to the research but not yet described in published literature, software must be made available to editors and reviewers. We strongly encourage code deposition in a community repository (e.g. GitHub). See the Nature Portfolio guidelines for submitting code & software for further information.

Data

Policy information about availability of data

All manuscripts must include a data availability statement. This statement should provide the following information, where applicable:

- Accession codes, unique identifiers, or web links for publicly available datasets
- A description of any restrictions on data availability
- For clinical datasets or third party data, please ensure that the statement adheres to our policy

The atomic coordinates were deposited in the RCSB Protein Data Bank (PDB) under accession codes 7UNQ (compact IF2-GDP, I-A), 7UNR (70S-IF2-GDP composite, I-A), 7UNT (compact IF2-GDP, I-B), 7UNU (70S-IF2-GDP composite, I-B), 7UNV (70S-IF2-GDPCP, II-A), 7UNW (70S-IF2-GDPCP, II-B), and 7UIU (IF2 N2 sub-domain). The cryo-EM maps have been deposited in the Electron Microscopy Data Bank (EMDB) under accession codes EMD-26628 (70S, I-A), EMD-26629 (compact IF2-GDP from focused refinement, I-A), EMD-26630 (70S-IF2-GDP composite, I-A), EMD-26631 (70S, I-B), EMD-26632 (compact IF2-GDP from focused refinement, I-B), EMD-26633 (70S-IF2-GDP composite, I-B), EMD-26634 (70S-IF2-GDPCP, II-A), EMD-26635 (70S-IF2-GDPCP, II-B), and EMD-26553 (IF2 N2 sub-domain from focused refinement). The unaligned multi-frame cryo-EM micrographs have been deposited in the Electron Microscopy Public Image Archive (EMPIAR) with the accession code EMPIAR-11012.

Field-specific reporting

Please select the one below that is the best fit for your research. If you are not sure, read the appropriate sections before making your selection.

☒ Life sciences ☐ Behavioural & social sciences ☐ Ecological, evolutionary & environmental sciences

For a reference copy of the document with all sections, see nature.com/documents/nr-reporting-summary-flat.pdf

Life sciences study design

All studies must disclose on these points even when the disclosure is negative.

Sample size	The cryo-EM dataset contains 8,056 micrographs, of which 7,259 were selected for further processing based on criteria set by CryoSPARC (v. 3.1.0). 878,576 particles were picked for 2D and 3D classification to achieve atomic resolution reconstructions as described in the Methods section and in Supplementary Fig. 3. Such dataset size yielded four class averages of the 70S ribosome initiation complex bound to initiation factor 2 (IF2), each containing at least 20,000 particles sufficient for 3D reconstructions at atomic resolution.
Data exclusions	All the data was included in the analysis. 2D and 3D classification of the particles was performed following criteria set by CryoSPARC (v. 3.1.0).
Replication	The cryo-EM experiment was repeated twice. We collected a second completely independent cryo-EM dataset of the same complex, which confirmed the findings.
Randomization	Computational unsupervised particle classification employs the maximum likelihood estimation method which includes randomization. Classifications were repeated multiple times varying the number of classes, which yielded similar particle distribution among the clusters.
Blinding	Blinding was not required because particle classification was performed in CryoSPARC (v. 3.1.0) which uses a maximum likelihood estimation method. Classification was repeated several times varying the number of classes, yielding similar particle distribution among the clusters.

Reporting for specific materials, systems and methods

We require information from authors about some types of materials, experimental systems and methods used in many studies. Here, indicate whether each material, system or method listed is relevant to your study. If you are not sure if a list item applies to your research, read the appropriate section before selecting a response.

Materials & experimental systems

n/a	Involved in the study	
☒	☐	Antibodies
☒	☐	Eukaryotic cell lines
☒	☐	Palaeontology and archaeology
☒	☐	Animals and other organisms
☒	☐	Human research participants
☒	☐	Clinical data
☒	☐	Dual use research of concern

Methods

n/a	Involved in the study	
☒	☐	ChIP-seq
☒	☐	Flow cytometry
☒	☐	MRI-based neuroimaging
